# The Effects of Propionate and Valerate on Insulin Responsiveness for Glucose Uptake in 3T3-L1 Adipocytes and C2C12 Myotubes via G Protein-Coupled Receptor 41

**DOI:** 10.1371/journal.pone.0095268

**Published:** 2014-04-18

**Authors:** Joo-Hui Han, In-Su Kim, Sang-Hyuk Jung, Sang-Gil Lee, Hwa-Young Son, Chang-Seon Myung

**Affiliations:** 1 Department of Pharmacology, Chungnam National University College of Pharmacy, Daejeon, Republic of Korea; 2 Department of Veterinary Pathology, Chungnam National University College of Veterinary Medicine, Daejeon, Republic of Korea; 3 Institute of Drug Research & Development, Chungnam National University, Daejeon, Republic of Korea; INSERM/UMR 1048, France

## Abstract

Since insulin resistance can lead to hyperglycemia, improving glucose uptake into target tissues is critical for regulating blood glucose levels. Among the free fatty acid receptor (FFAR) family of G protein-coupled receptors, GPR41 is known to be the Gα_i/o_-coupled receptor for short-chain fatty acids (SCFAs) such as propionic acid (C3) and valeric acid (C5). This study aimed to investigate the role of GPR41 in modulating basal and insulin-stimulated glucose uptake in insulin-sensitive cells including adipocytes and skeletal muscle cells. Expression of GPR41 mRNA and protein was increased with maximal expression at differentiation day 8 for 3T3-L1 adipocytes and day 6 for C2C12 myotubes. GPR41 protein was also expressed in adipose tissues and skeletal muscle. After analyzing dose-response relationship, 300 µM propionic acid or 500 µM valeric acid for 30 min incubation was used for the measurement of glucose uptake. Both propionic acid and valeric acid increased insulin-stimulated glucose uptake in 3T3-L1 adipocyte, which did not occur in cells transfected with siRNA for GPR41 (siGPR41). In C2C12 myotubes, these SCFAs increased basal glucose uptake, but did not potentiate insulin-stimulated glucose uptake, and siGPR41 treatment reduced valerate-stimulated basal glucose uptake. Therefore, these findings indicate that GPR41 plays a role in insulin responsiveness enhanced by both propionic and valeric acids on glucose uptake in 3T3-L1 adipocytes and C2C12 myotubes, and in valerate-induced increase in basal glucose uptake in C2C12 myotubes.

## Introduction

Diabetes mellitus is the most common metabolic disease and its increased prevalence has raised attention as a worldwide public health problem. More than 80% of diabetes patients suffer from type 2 diabetes [1]. Type 2 diabetes does not involve a lack of insulin secretion but rather is characterized by insulin resistance, a state in which insulin has a reduced ability to mediate glucose homeostasis in its major target tissues, such as skeletal muscle, adipose tissue, and liver [2]. Moreover, insulin resistance is a cause of many related complications, including hyperglycemia, dyslipidemia, hypertension, and atherosclerosis [3]. Thus, the improvement of insulin resistance can be an effective intervention in type 2 diabetes and its related complications.

Glucose is one of the major sources of energy for the human body, and blood glucose concentrations are regulated within a narrow range of 5–6 mM, predominantly by insulin [4], [5]. The first step by which insulin increases energy storage or utilization involves the regulated transport of glucose into the cell [6]. Glucose transport in insulin-sensitive tissues (skeletal muscle and adipose tissue) is a control point for the regulation of blood glucose levels, and a possible target for the derangement of glucose homeostasis in certain disease states, such as type 2 diabetes [7].

G protein-coupled receptors (GPCRs) are involved in many physiological processes, such as the biological actions of insulin, and are activated by a variety of endogenous ligands, such as hormones, neurotransmitters, peptides, proteins, steroids, fatty acids, and other lipids [8], [9]. Fatty acids have long been recognized for the variety of their activities in the body, but these were thought to be mediated by effects on cellular metabolism [10]. Recently, several groups identified five orphan receptors that can be activated by free fatty acids (FFAs), which function on the cell surface and play significant roles [11]. Long-chain fatty acids (with more than 12 carbon atoms) are specific agonists of GPR40 (free fatty acid receptor 1, FFAR1) and GPR120, and medium-chain fatty acids (with 7–12 carbon atoms) of GPR84. Short-chain fatty acids (SCFAs), consisting of 1–6 carbon atoms, can activate GPR41 (FFAR3) and GPR43 (FFAR2). Both GPR40 and GPR120 have been reported to signal via Gα_q_, GPR41 via Gα_i/o_, and GPR43 through both Gα_q_ and Gα_i/o_ [12].

Before ‘deorphanizing’ GPR41, Green et al. reported that after down-regulation of Gα_i_ subunits, insulin resistance developed in adipocytes [13]. GPR41 was first deorphanized by two groups in 2003 [14], [15]. The expression of GPR41 in both human and mouse adipose tissue has been detected, and it was reported that SCFAs promote the secretion of leptin, a hormone regulating energy intake and expenditure, via GPR41 [16]. However, another research group did not detect GPR41 expression in murine adipose tissue or 3T3-L1 adipocytes [17]. Thus, the expression of GPR41 in adipose tissue remains controversial. In human skeletal muscle, GPR41 mRNA was detected and the amount was lower than that of adipose tissue [14]. SCFA-bound Gα_i/o_-coupled GPR41 activation resulted in decreased cAMP production and activation of the extracellular signal-regulated kinase (ERK) cascade [15], [18]. Other physiological functions of GPR41 remain to be explored.

The aim of this study was to investigate the effects of SCFAs such as propionic acid (C3) and valeric acid (C5) on insulin sensitivity via GPR41. Using differentiated 3T3-L1 adipocytes and C2C12 skeletal muscle cells, we demonstrate that both propionic acid and valeric acid increase glucose uptake in these cells via, at least in part, GPR41, suggesting GPR41 to be a potential target for the regulation of blood glucose levels.

## Materials and Methods

### 1. Materials

Dulbecco's modified Eagle's medium (DMEM), fetal bovine serum (FBS), bovine calf serum, phosphate-buffered saline (PBS), and trypsin-EDTA were from Gibco BRL (Grand Island, NE, USA). Penicillin/streptomycin was from Thermo Scientific (Rockford, IL, USA). Propionic acid, valeric acid, 2-deoxy-D-glucose, dexamethasone, 3-isobutyl-1-methylxanthine (IBMX), insulin, and 3-(4,5-dimethylthiazol-2-yl)-2,5,-diphenyltetrazolium bromide (MTT) were from Sigma-Aldrich (St. Louis, MO, USA). 2-Deoxy-[^3^H]-glucose was obtained from PerkinElmer Life Sciences (Boston, MA, USA). Rosiglitazone was purchased from Masung & Co., Ltd (Seoul, Korea). The anti-GPR41 (H-100) antibody was from Santa Cruz Biotechnology Inc. (Santa Cruz, CA, USA). The anti-PPARγ (D69) antibody was from Cell Signaling Technology, Inc. (Beverly, MA, USA). Anti-β-actin and goat anti-rabbit antibodies were from Abfrontier (Geumcheon, Seoul, Korea). The anti-myosin heavy chain (MHC; MF 20) antibody was obtained from the Development Studies Hybridoma Bank (Iowa City, IA, USA). Horseradish peroxidase-conjugated secondary antibodies (peroxidase anti-rabbit IgG produced in goat, #PI-1000; peroxidase anti-mouse IgG produced in horse, #PI-2000) to detect the primary antibodies were purchased from Vector Laboratories Inc. (Burlingame, CA, USA). Other chemicals were of analytical grade.

### 2. Cell culture and differentiation

3T3-L1 preadipocytes were grown in DMEM with 10% bovine calf serum at 37°C in a 5% CO_2_ atmosphere. Two days after 3T3-L1 cells had reached confluence (designated day 0), differentiation was induced by treating the cells with 10% FBS, 0.5 mM IBMX, 1 µM dexamethasone, and 1 µg/mL insulin for 3 days. The medium was replaced with DMEM containing 10% FBS and 1 µg/mL insulin for the following 2 days, and the cells were then maintained in DMEM with 10% FBS, which was replaced every 2 days until day 8 [19]. C2C12 myoblasts were grown in DMEM with 10% FBS at 37°C in a 5% CO_2_ atmosphere. Confluent myoblasts (designated day 0) were cultured in DMEM containing 1% FBS to differentiate C2C12. Then, medium was replaced every day until day 6 [20].

### 3. Tissue isolation

Eight-week-old male C57BL/6 mice were purchased from Charles River Japan, Inc. Animals were housed four in a cage and raised in a room of controlled temperature (22±2°C), humidity (50±5%), and lighting (12/12-h dark-light cycle, lights on 6:00 a.m.). After a 1-week acclimatization period, animals were sacrificed by decapitation. White adipose tissues were separated from epididymal and mesenteric fat sites, and brown adipose tissues from retroperitoneal fat sites. Skeletal muscle tissues from thigh sites, and liver were collected. Each tissue per animal was separated, rinsed by phosphate-buffered saline (PBS) and stored in refrigerator until use for western blotting. All experimental protocols were approved and performed according to the Guide for the National Institutes of Health Guide for the Care and Use of Laboratory Animals as approved by Chungnam National University Animal Care and Use Committee.

### 4. Cell viability assay

Cytotoxicity was determined using the MTT reduction assay. 3T3-L1 preadipocytes or C2C12 myoblasts were seeded into 96-well culture plates at 4×10^3^/well and then cultured in growth medium at 37°C for 24 h. When cells reached 70% confluence, the medium was replaced with serum-free medium containing various concentrations of propionic acid or valeric acid. Cells were incubated for 24 h and MTT reagent (5 mg/mL) was added to each well. After 4 h, formazan crystals formed in the actively metabolizing cells were extracted with dimethyl sulfoxide (DMSO), and the absorbance at 570 nm was measured using spectrophotometer (Tecan Group Ltd., Männedorf, Switzerland). Differentiated 3T3-L1 adipocytes (day 7) or C2C12 myotubes (day 5) were also treated with various concentration of propionic acid or valeric acid and incubated for 24 h. After adding MTT reagent for 2 h (3T3-L1 adipocytes) or 3 h (C2C12 myotubes), cells were treated with DMSO and the absorbance was measured.

### 5. Glucose uptake measurement

A glucose uptake assay was performed as described previously, with slight modification [21]. Briefly, 3T3-L1 adipocytes or C2C12 myotubes were serum-starved in DMEM, and the cells were then incubated in Krebs-Ringer phosphate-HEPES (KRPH) buffer (10 mM HEPES, pH 7.4, 136 mM NaCl, 4.7 mM KCl, 1 mM MgSO_4_, 1 mM CaCl_2_, 10 mM phosphate buffer). Various concentrations of propionic acid or valeric acid were added to the medium alone (basal glucose uptake) for different time points or followed by insulin (100 nM, insulin-stimulated glucose uptake) for 30 min. Glucose uptake was initiated by the addition of 2-deoxy-[^3^H]-glucose (0.1 µCi/mL in 3T3-L1 adipocytes or 0.5 µCi/mL in C2C12 myotubes) with 100 µM 2-deoxy-D-glucose in each well. After 10 min, cells were washed three times with ice-cold PBS and lysed with 0.1% sodium dodecyl sulfate (SDS) and 0.5 M NaOH. The radioactivity was determined using liquid scintillation counting in a β-counter and normalized according to the total protein level. Nonspecific uptake was determined in the presence of 10 µM cytochalasin B. Rosiglitazone (10 µM) treatment for 48 h was used as a positive control.

### 6. Quantification of gene expression levels by quantitative real-time PCR (qPCR)

Total RNA was isolated using the Total RNA Extraction Kit (Real-Biotech Co., Minsheng Rd., Taiwan). All RNAs were treated with DNaseI (RNase-free; Takara, Japan) and equal amounts of total RNA were reverse-transcribed to cDNA using the AccuPower CycleScript RT PreMix (dN12) (Bioneer, Daejeon, Korea). qPCR was performed with SYBR Green qPCR Premix (Toyobo, Japan) and specific primer pairs using the Exicycler 96 (Bioneer). The sense and antisense primers for β-actin (NM_001101) were 5′-TCC ATC ATG AAG TGT GAC GT-3′ and 5′-GCT CAG GAG GAG CAA TGA T-3′; for GPR41-mouse (NM_001033316.2) 5′-TGT CCA ATA CTC TGC ATC TGT G-3′ and 5′-AGG TCC GAA ATG GTC AGG TT-3′, respectively. mRNA expression levels are presented as the threshold cycle (Ct) values, normalized to the β-actin mRNA value.

### 7. Western blotting

Total protein extracts were prepared from tissues or scraped cells using lysis buffer (Pro-prep Protein extraction solution, Intron Biotechnology, Seoul, Korea). Lysates were incubated at 4°C for 30 min and then centrifuged at 13,000 rpm for 10 min at 4°C to remove insoluble materials. The protein concentrations were determined using a BCA protein assay kit (Pierce, Rockford, IL, USA). For each blot, equal amounts of cell lysates (15–20 µg protein) were separated by SDS-polyacrylamide gel electrophoresis (SDS-PAGE, 7.5–12.5%) and transferred onto polyvinylidene fluoride (PVDF) membranes (ATTO Corp., Tokyo, Japan) at 200 mA for 2 h. The blots were blocked with 5% BSA in TBS-T and incubated with primary antibodies overnight at 4°C and subsequently with a secondary antibody. Protein bands were detected using ECL kit (Abfrontier, Korea) and the intensities of bands were quantified using the Quantity One software (Bio-Rad, Hercules, CA, USA). The detected proteins were normalized to levels of β-actin in cell lines and GAPDH (glyceraldehyde 3-phosphate dehydrogenase) in tissues.

### 8. siRNA transfection

The GPR41 siRNA oligonucleotide (target accession no. NM_001033316.1) and negative siRNA oligonucleotide were synthesized by Bioneer Corp. (Daejeon, Korea). The target sequence of the siRNA for GPR41 (siGPR41) used was sense 5′-CAC UGU AGU GUG GUU UAC A(dTdT)-3′ and antisense 5′-UGU AAA CCA CAC UAC AGU G(dTdT)-3′. The siRNA oligonucleotide was transfected into 3T3-L1 adipocytes and C2C12 myotubes using Lipofectamine RNAiMAX (Invitrogen, Paisley, UK) according to the manufacturer's protocol. Final concentrations of 100 nM siGPR41 oligonucleotide were selected for both 3T3-L1 adipocytes and C2C12 myotubes and were transfected into the cells for 48 h before the treatment with propionic acid or valeric acid and assays. The ability of the siRNA oligonucleotide to knock down GPR41 expression was analyzed by Western blotting of whole cell extracts.

### 9. Statistical analysis

All values are expressed as means ± SEM, and the significance of differences between groups was tested using a two-tailed unpaired Student's *t*-test. Differences between more than two groups were tested using Dunnett's multiple comparison test using the GraphPad software (San Diego, CA, USA). A *P* value <0.05 was considered to indicate statistical significance.

## Results

### 1. GPR41 Expression

**Figure 1:**
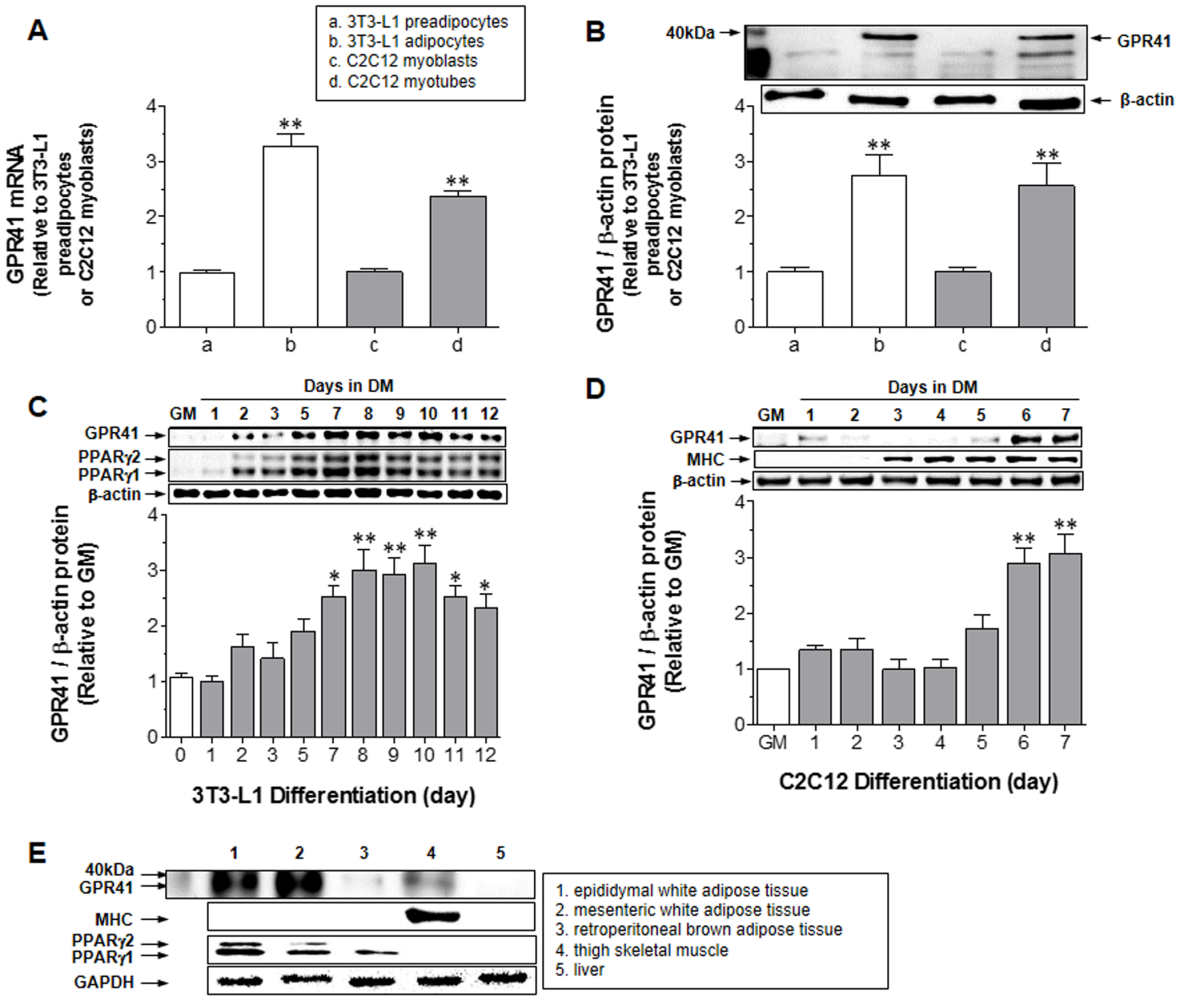
GPR41 expression. GPR41 mRNA (A) and protein (B) levels were assayed in 3T3-L1 preadipocytes, differentiated adipocytes on day 8, C2C12 myoblasts, and differentiated myotubes on day 6 by quantitative real-time PCR and immunoblotting, as described in the Methods. Bar graph expresses relative values to 3T3-L1 preadipocytes or C2C12 myoblasts. **P<0.01, vs. 3T3-L1 preadipocytes or C2C12 myoblasts. GPR41 protein levels were measured during differentiation of 3T3-L1 adipocytes (C) and C2C12 myotubes (D). Day 0 was set for adipocyte differentiation by growing 3T3-L1 preadipocytes to 2 days post-confluence in growth medium (GM) and for myotubes by growing C2C12 myoblasts to confluence in GM. **P<0.01 and *P<0.05, vs. GM. Gel images are representative of the three experiments. All values expressed in the bar graphs are means ± SEM and the average of three similar, independent experiments, each performed in triplicate. The expression level of GPR41 protein was measured in five different tissues of nine-week-old male C57BL/6 mice (E) as described in the Methods. Gel images shown are representative of four similar experiments with different protein extracts of five tissues from four mice (n = 4). Total GPR41 protein was normalized to the β-actin level in cell lines and GAPDH level in tissues.

To confirm the presence of GPR41, mRNA and protein expression levels of GPR41 were measured in 3T3-L1 preadipocytes, differentiated 3T3-L1 adipocytes, C2C12 myoblasts, and differentiated C2C12 myotubes. Both 3T3-L1 adipocytes and C2C12 myotubes expressed significantly GPR41 mRNA (Fig. 1A) and protein (Fig. 1B) at high levels. To establish the optimal time for differentiation of 3T3-L1 adipocytes and C2C12 myotubes with the highest level of GPR41 protein expression, GPR41 protein expression levels on each differentiation day were measured. The highest level of GPR41 protein expression was at days 8–10 in adipocytes (Fig. 1C) and at days 6–7 in myotubes (Fig. 1D). The expression patterns of differentiation markers – peroxisome proliferator-activated receptor γ (PPARγ) for adipocytes and myosin heavy chain (MHC) for myotubes – were similar. Thus, adipocytes at differentiation day 8 and myotubes at day 6 were used in the following experiments. Neither mRNA nor protein expression of GPR41 was detected in HepG2 (human hepatocellular carcinoma) cells (data not shown). Figure 1E showed that GPR41 protein was mostly expressed in epididymal and mesenteric white fat tissues, and thigh skeletal muscle. Similarly, PPARγ for adipose tissues and MHC for skeletal muscle were coincidentally expressed in these tissues. No significant detection of GPR41 protein expression was found in retroperitoneal brown adipose tissue and liver. Thus, GPR41 protein is expressed in two major insulin-sensitive tissues of mice, regarding glucose transport: white adipose tissues and skeletal muscle.

### 2. Cytotoxicity of SCFAs

**Figure 2:**
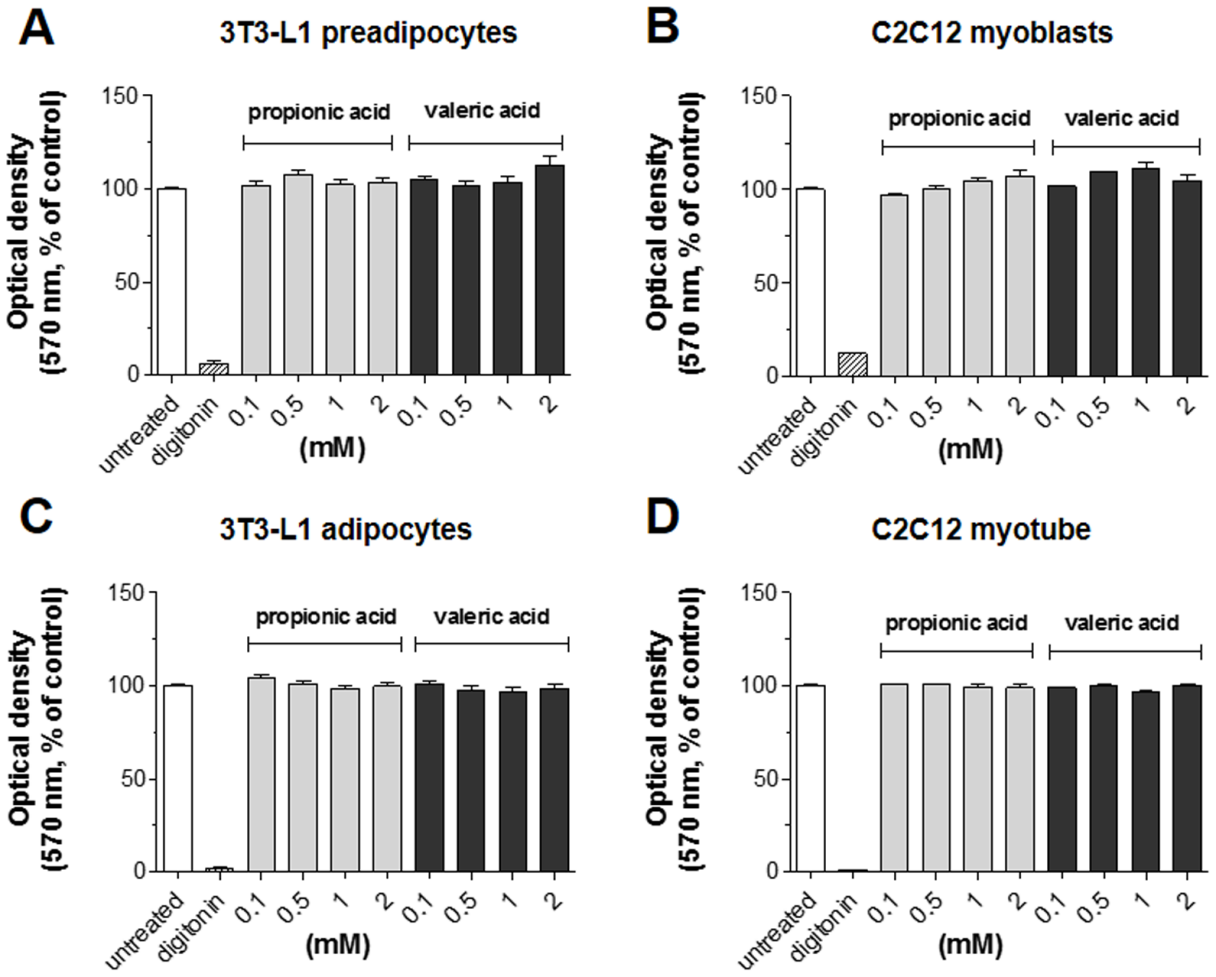
Cytotoxicity of propionic acid and valeric acid in both non-differentiated and differentiated 3T3-L1 and C2C12 cells. 3T3-L1 preadipocytes (A), C2C12 myoblasts (B), 3T3-L1 adipocytes (C) and C2C12 myotubes (D) were exposed to the indicated concentrations of propionic acid or valeric acid for 24 h, and cell viability was measured at 570 nm using the MTT assay as described in the Methods. Digitonin (100 µg/mL) was used as the positive control. Results are the means ± SEM of three similar independent experiments, each performed in triplicate.

To examine whether the administration of GPR41 agonists was cytotoxic to cells, 3T3-L1 preadipocytes, C2C12 myoblasts, differentiated 3T3-L1 adipocytes and C2C12 myotubes were incubated with various concentrations of propionic acid or valeric acid up to 2 mM for 24 h and the MTT assay was performed. Figure 2 presented that neither agonist, at any concentration, affected the viability of all cells tested. Thus, the GPR41 agonists – propionic acid and valeric acid – were not cytotoxic to these cells. Digitonin was used as a positive control for cytotoxicity.

### 3. Effect of SCFAs on glucose uptake in 3T3-L1 adipocytes and C2C12 myotubes

**Figure 3:**
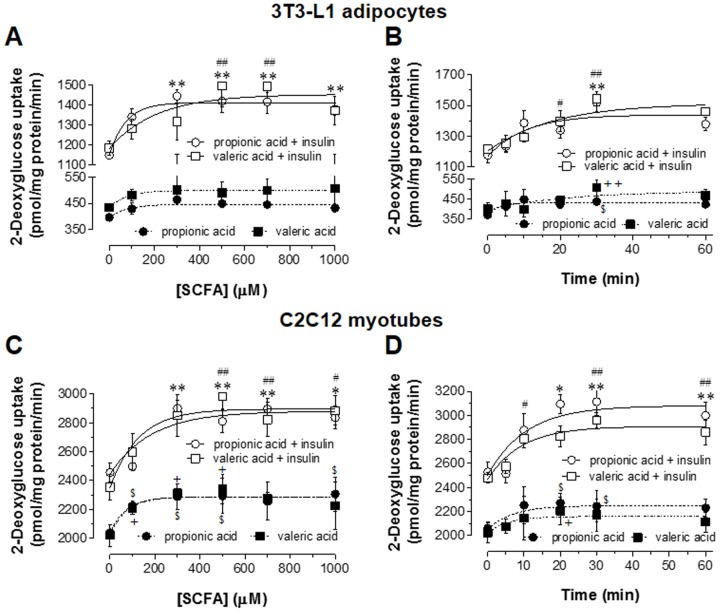
Dose-response relationship between SCFAs and glucose uptake in 3T3-L1 adipocytes and C2C12 myotubes, and time-course analysis. For the determination of dose-response relationship, 3T3-L1 adipocytes (A) or C2C12 myotubes (C) were starved and treated with various concentrations of propionic acid or valeric acid for 30 min in the absence or presence of insulin (100 nM) in KRPH buffer. For the analysis of time-course, 3T3-L1 adipocytes (B) and C2C12 myotubes (D) were treated with 300 µM propionic acid or 500 µM valeric acid for the indicated time in the absence or presence of insulin in KRPH buffer. After adding 2-deoxy-[^3^H]-glucose for 10 min, glucose uptake was measured in the cell lysates as described in the Methods. Results are the means ± SEM of three similar independent experiments, each performed in quadruplicate. *P<0.05 and **P<0.01 for propionic acid, and ^#^ *P*<0.05 and ^##^ *P*<0.01 for valeric acid, vs. insulin-stimulated glucose uptake with no SCFA treatment. ^$^ *P*<0.05 for propionic acid, ^+^ *P*<0.05 and ^++^ *P*<0.01 for valeric acid, vs. basal glucose uptake with no SCFA treatment.

To investigate the effect of SCFAs on glucose uptake, dose-response relationship in 3T3-L1 adipocytes and C2C12 myotubes treated with various concentrations of propionic acid or valeric acid for 30 min was examined and time course with fixed concentrations of propionic acid or valeric acid was analyzed. In 3T3-L1 adipocytes, insulin-stimulated glucose uptake was increased as concentrations of propionic acid and valeric acid were increased (Fig. 3A). However, basal glucose uptake by either SCFA was not significantly increased. In C2C12 myotubes, these two SCFAs significantly increased both insulin-stimulated and basal glucose uptake (Fig. 3C). The maximal effects on insulin-stimulated glucose uptake in both 3T3-L1 adipocytes and C2C12 myotubes were reached at 300 µM propionic acid and 500 µM valeric acid, respectively. The responses by higher concentrations of propionic acid and valeric acid reached to plateau. In basal state, both propionic acid (100, 300, 500, 1000 µM) and valeric acid (100, 300, 500 µM) increased significantly glucose uptake in C2C12 myotubes (*P*<0.05). Figures 3B and 3D show that insulin-stimulated glucose uptake was significantly increased up to a maximal plateau after 30 min incubation with 300 µM propionic acid and 500 µM valeric acid. In case of basal glucose uptake, significant response was reached to plateau within 30 min in both 3T3-L1 adipocytes and C2C12 myotubes. Thus, 3T3-L1 adipocytes and C2C12 myotubes were treated with 300 µM propionic acid or 500 µM valeric acid for 30 min in this study.

**Figure 4:**
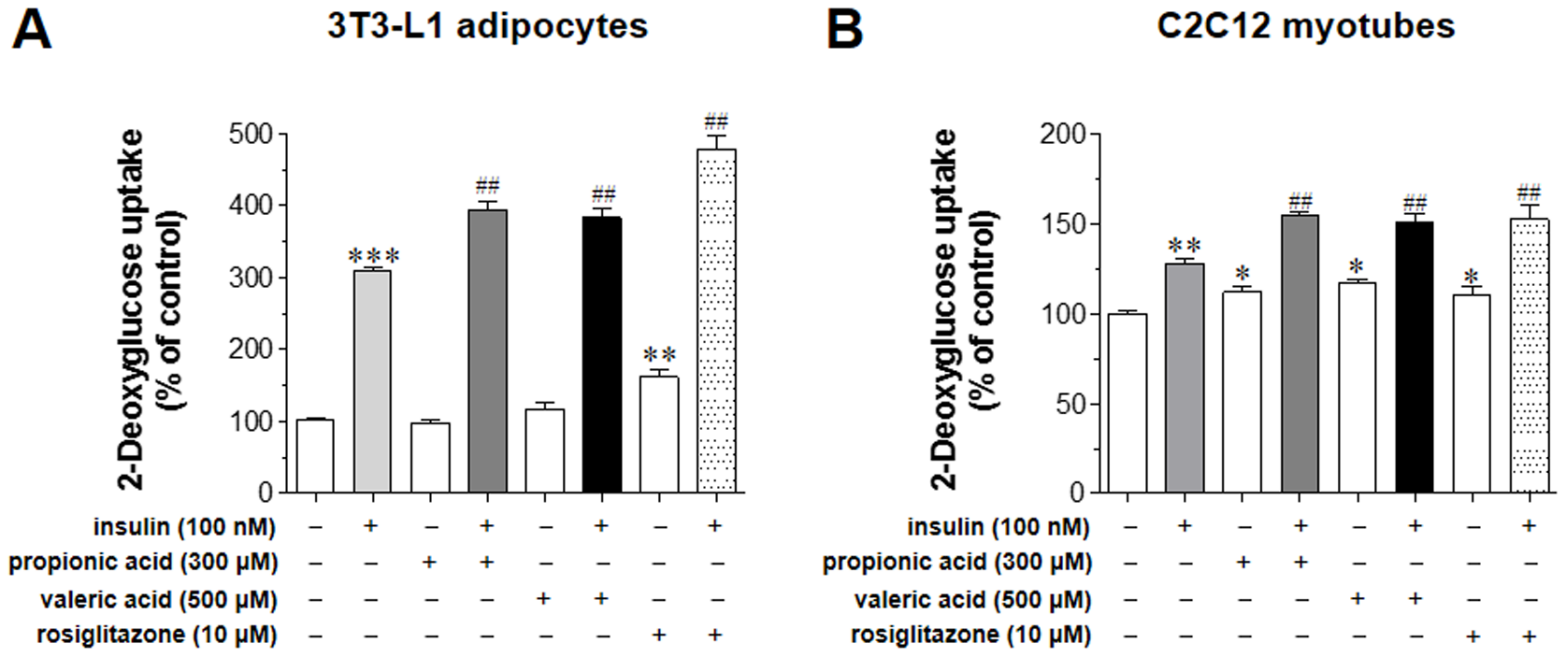
Effect of SCFAs on basal and insulin-stimulated glucose uptake in 3T3-L1 adipocytes and C2C12 myotubes. The activity of 300 µM propionic acid and 500 µM valeric acid to increase basal and insulin-stimulated glucose uptake was measured in 3T3-L1 adipocytes (A) and C2C12 myotubes (B) as described in the Methods. These cells were treated with rosiglitazone (10 µM), as a positive control, for 48 h, and glucose uptake was measured in the cell lysates. Results are the means ± SEM of three similar independent experiments, each performed in quadruplicate. *P<0.05, **P<0.01, ***P<0.001, vs. basal glucose uptake (no insulin stimulation), ^##^ *P*<0.01, vs. insulin-stimulated glucose uptake with no SCFA treatment.

In 3T3-L1 adipocytes (Fig. 4A), control (no insulin and no SCFAs, 1^st^ open bar) was set as 100% and insulin significantly increased glucose uptake by 207.1% [difference (Δ) to control]. Both 300 µM propionic acid and 500 µM valeric acid increased significantly insulin-stimulated glucose uptake by 85.1% (Δ to insulin-treated) and 74.8%, respectively. A PPARγ agonist, rosiglitazone as a positive control [21]–[23], increased significantly both basal by 60.0% and insulin-stimulated glucose uptake by 170.3%. However, in the case of rosiglitazone, the increasing rate of glucose uptake merely by insulin was about 110.3% (Δ between insulin-stimulated and basal glucose uptake). In C2C12 myotubes (Fig. 4B), insulin increased significantly glucose uptake by 28.3%, and both 300 µM propionic acid and 500 µM valeric acid increased insulin-stimulated glucose uptake to 26.4% and 23.3%, respectively. Interestingly, 300 µM propionic acid and 500 µM valeric acid also significantly increased basal glucose uptake by 12.4% (*P*<0.05) and 16.3% (*P*<0.05), respectively. These results indicate that the increasing amount of insulin-stimulated glucose uptake by both SCFAs seems to be owing to the increment of basal glucose uptake. Thus, increasing rates of 300 µM propionic acid and 500 µM valeric acid on glucose uptake exclusively by insulin in C2C12 myotubes were 14.0% (Δ between insulin-stimulated and basal glucose uptake) and 7.0%, respectively, which were not statistically significant. Rosiglitazone (10 µM) increased both insulin-stimulated and basal glucose to 24.7% (*P*<0.01) and 10.6% (*P*<0.05), respectively, and pure increasing rate of glucose uptake by insulin was 14.1% (*P*<0.05). Taken together, both 300 µM propionic acid and 500 µM valeric acid increased significantly insulin-stimulated glucose uptake in 3T3-L1 adipocytes, and these SCFAs did not potentiate insulin-stimulated glucose uptake in C2C12 myotubes.

### 4. Effect of SCFAs on glucose uptake in 3T3-L1 adipocytes and C2C12 myotubes treated with GPR41 siRNA

**Figure 5:**
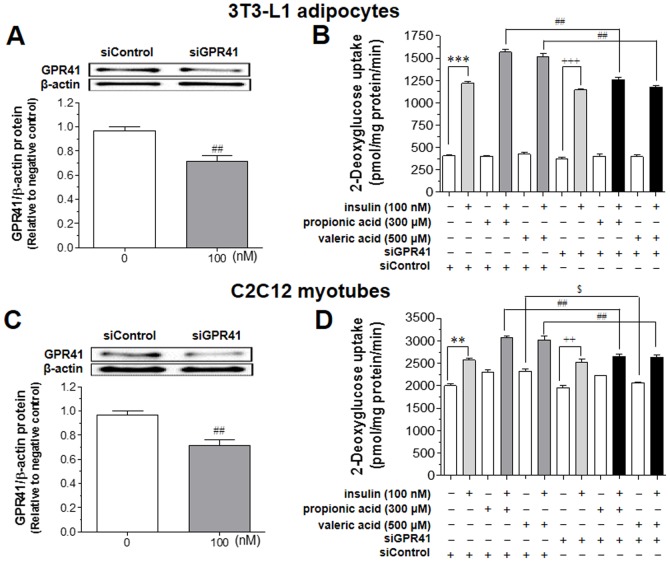
Effects of siRNA for GPR41 on SCFA-induced rise in insulin-stimulated glucose uptake in 3T3-L1 adipocytes and C2C12 myotubes. After confirming the expression of GPR41 protein expression by transfecting with siRNA for GPR41 (siGPR41,100 nM) using Lipofectamine RNAiMAX for 48 h in 3T3-L1 adipocytes (A) or C2C12 myotubes (C), cells were treated with 300 µM propionic acid or 500 µM valeric acid for 30 min in the absence or presence of insulin (100 nM) in KRPH buffer. Glucose uptake was measured in the lysates of 3T3-L1 adipocytes (B) or C2C12 myotubes (D) as described in the Methods. Results are the means ± SEM of three similar independent experiments, each performed in quadruplicate. **P<0.01 and ***P<0.001, vs. basal glucose uptake with siControl (negative control siRNA). ^++^ *P*<0.01 and ^+++^ *P*<0.001 vs. basal glucose uptake with siGPR41, ^##^ *P*<0.01 vs. insulin-stimulated glucose uptake with SCFAs and siControl.

To examine that the increased effect of insulin-stimulated glucose uptake by SCFAs is mediated via GPR41, the GPR41 gene was down regulated in 3T3-L1 adipocytes and C2C12 myotubes using GPR41 siRNA oligonucleotide (siGPR41). siGPR41 transfection reduced GPR41 protein expression by ∼30% in both 3T3-L1 adipocytes (Fig. 5A) and C2C12 myotubes (Fig. 5C). The SCFA-induced increase in glucose uptake was reduced significantly by siGPR41 treatment in both 3T3-L1 adipocytes (Fig. 5B) and C2C12 myotubes (Fig. 5D).

Under siControl (negative control siRNA) treatment in 3T3-L1 adipocytes, untreated group (no insulin, no SCFAs, and no siGPR41) showed basal glucose uptake (405.86±38.04 pmol/mg protein/min), and insulin treatment increased to 1218.20±51.66. As expected, siControl did not produce any reducing effect of SCFAs on insulin-stimulated glucose uptake in 3T3-L1 adipocytes. Under siGPR41 treatment in differentiated adipocytes, basal glucose uptake was 376.0±40.85 and insulin increased significantly to 1146.0±35.33, indicating that glucose uptake was not affected by siGPR41 treatment. In 3T3-L1 adipocytes, siGPR41 treatment reduced significantly 300 µM propionic acid-induced increase in insulin-stimulated glucose uptake from 1566.22±89.47 to 1261.95±52.85, and 500 µM valeric acid from 1510.52±95.75 to 1175.05±41.71, respectively.

Similarly, insulin treatment in C2C12 myotubes stimulated significantly glucose uptake from 2000.00±136.71 (pmol/mg protein/min) to 2567.94±150.90. Increase in insulin-stimulated glucose uptake by 300 µM propionic acid was reduced significantly by treatment with siGPR41 from 3070.90±93.97 to 2656.01±109.99. Cells treated with 500 µM valeric acid and siGPR41 also reduced significantly from 3025.43±242.93 to 2637.80±103.61. As mentioned above, increasing effect of both propionic acid and valeric acid in C2C12 myotubes seemed to be due to the increase in basal glucose uptake. siGPR41 treatment in valeric acid-stimulated C2C12 myotubes reduced significantly basal glucose uptake from 2327.10±78.80 to 2058.95±47.42 (*P*<0.05), although propionic acid-induced increase in basal glucose uptake was not affected by siGPR41 treatment. Thus, these results indicate that the increase of insulin responsiveness of glucose uptake induced by propionic and valeric acids occurs via, at least in part, GPR41 in 3T3-L1 adipocytes and C2C12 myotubes, and valeric acid-mediated basal glucose uptake is associated with GPR41 in C2C12 myotubes.

## Discussion

This study has two major findings: (1) GPR41, a receptor for SCFAs such as propionic acid and valeric acid, was expressed in insulin-sensitive cell lines including 3T3-L1 adipocytes and C2C12 myotubes and tissues including adipose tissues and skeletal muscle, and (2) both propionic acid and valeric acid increased insulin-stimulated glucose uptake in 3T3-L1 adipocytes and basal glucose uptake in C2C12 myotubes via, at least in part, GPR41. This is the first report that propionic acid and valeric acid ameliorate insulin sensitivity via, at least in part, GPR41 by stimulating insulin-induced glucose uptake into adipocytes and basal glucose uptake into skeletal muscle cells. Our observations suggest that GPR41 may be a new molecular target to control high blood glucose level-associated disease states, such as type 2 diabetes.

Type 2 diabetes is characterized by insulin resistance, in which normal circulating concentrations of insulin are unable to regulate glucose levels in target tissues, such as fat, muscle, and liver [24]. Thus, finding molecular targets to diminish insulin resistance is important for the management of type 2 diabetes and related complications. Recently, several FFARs have been suggested as molecular targets for stimulation of insulin secretion. GPR40 is highly expressed in pancreatic β-cells and has been implicated in glucose-stimulated insulin secretion [25]. GPR119 is distributed in pancreatic β-cells and enteroendocrine L-cells, and some studies have suggested that GPR119 enhances insulin secretion directly in pancreatic β-cells, and increases insulin sensitivity indirectly via augmenting glucose-induced glucagon-like peptide-1 (GLP-1) secretion [26], [27]. Similarly, GPR120 has been reported to be associated with release of GLP-1 and repression of macrophage-induced inflammation [28], [29]. GPR41 is expressed abundantly in adipose tissue and mediates the stimulation of leptin production in adipocytes by GPR41 agonists, such as SCFAs [16]. A recent report revealed that butyrate suppressed lipolysis effects in 3T3-L1 adipocytes via GPR41 [30]. The results in the present study demonstrate that GPR41 has effects on insulin-stimulated glucose uptake increases in 3T3-L1 adipocytes and basal glucose uptake in C2C12 myotubes by SCFAs, propionic acid and valeric acid.

GPR41 expression in adipose tissue is controversial. However, our data (Fig. 1) demonstrate the detection of mRNA and protein of GPR41 in differentiated 3T3-L1 adipocytes and C2C12 myotubes. Furthermore, the expression patterns correspond with the differentiation periods, supported by the similar expression patterns of differentiation markers: PPARγ for 3T3-L1 adipocytes and MHC for C2C12 myotubes. Accordance with expression profile in cell lines, GPR41 protein expression was confirmed in insulin-sensitive tissues such as adipose tissues and skeletal muscle (Fig. 1E).

SCFAs are known agonists of both GPR41 (FFAR3) and GPR43 (FFAR2). The receptor specificity of SCFAs is determined by carbon chain length. Fatty acids with C3–C5 chain length are more potent agonists of GPR41, whereas those with a C2–C3 chain length are more potent agonists of GPR43 [31]. Our data (Fig. 4A) showed that in 3T3-L1 adipocytes, propionic acid (C3) increased insulin-stimulated glucose uptake, not basal, significantly by 85.1% and valeric acid (C5) by 74.8%. Thus, although propionic acid is stronger than valeric acid, both SCFAs increased significantly insulin-stimulated glucose uptake in 3T3-L1 adipocytes. On the contrary, both propionic and valeric acids did not potentiate insulin-stimulated glucose uptake in C2C12 myotubes due to significant increase in basal glucose uptake (Fig. 4B). Interestingly, SCFAs-induced stimulation of glucose uptake in both cell types was blocked by transfection with GPR41 siRNA, indicating that the effects of these two SCFAs on glucose uptake were, at least in part, GPR41-mediated. siGPR41 treatment suppressed the stimulation of basal glucose uptake induced by valeric acid, but not by propionic acid in C2C12 myotubes. This observation may suggest that valeric acid is more GPR41-specific to increase basal glucose uptake than propionic acid, however, this issue needs to study further. Thus, our data suggest that SCFAs acting via GPR41 have an ‘insulin-sensitizing’ effect in adipocytes, whereas these have an ‘insulin-like’ effect in skeletal muscle cells.

Our dose-response analyses showed that maximal effects on glucose uptake were obtained with 300 µM propionic acid and 500 µM valeric acid (Fig. 3). It is reported that the principal SCFAs including propionic acid are the predominant luminal anions in colonic fluid, with a normal concentration range of 70–100 mM and a relative ratio of 60 acetate:25 propionate:15 butyrate [32]. After transferring to blood stream, the blood concentration of propionic acid was reported to around 3.8–4.6 µM in humans [33]. Although the concentrations of propionic acid and valeric acid tested in this study may not be relevant to blood concentration of propionic acid reported, this study was performed in differentiated cell lines of adipose tissue and skeletal muscles. Thus, future study with using in vivo system can elucidate this issue.

It remains unclear whether the effects of propionic acid and valeric acid on basal and insulin-stimulated glucose uptake were mediated only via GPR41, or also by GPR43. It has been reported that GPR43 is expressed in adipose tissue and 3T3-L1 adipocytes, and that acetate and propionic acid stimulate adipogenesis, upregulate PPARγ, and inhibit isoproterenol-induced lipolysis via GPR43 [17]. These effects could also be stimulated via GPR41, as reported elsewhere [30]. Further studies are needed to clarify the roles and interactions of GPR41 and GPR43 in increasing basal and insulin-stimulated glucose uptake, which will contribute to our understanding of glucose regulation.

Involvement of ERK1/2 signaling for insulin sensitivity is controversial. In cardiomyocytes, oxidative stress induced by chronic treatment with H_2_O_2_ activated ERK1/2 signaling, and then led to insulin resistance [34]. Moreover, ERK1/2 activation induced by angiotensin II suppressed insulin sensitivity by inhibiting the insulin-induced insulin receptor substrate 1 (IRS-1) tyrosine phosphorylation and glucose uptake in vascular smooth muscle cells [35]. In contrast, palmitate stimulates glucose uptake in skeletal muscle cells via activation of the phosphoinositide 3-kinase (PI3K)-AMP-activated protein kinase (AMPK)-Akt and PI3K-ERK1/2 signaling [36]. Other reports present that inhibition of ERK1/2 activation by treating with ERK pathway inhibitor, PD184352, has no effect on insulin-stimulated glucose uptake in 3T3-L1 adipocytes [37]. Until now, it seems that ERK1/2 signaling may have different roles according to cell and tissue types. Thus, the direct coupling glucose uptake to ERK1/2 regulation by Gα_i/o_-coupled GPR41 agonists needs to be investigated in 3T3-L1 adipocytes and C2C12 myotubes in the future.

In conclusion, both propionic and valeric acids increased significantly insulin-stimulated glucose uptake in 3T3-L1 adipocytes and basal glucose uptake in C2C12 myotubes. These effects of both SCFAs known to be GPR41 agonists on glucose uptake are mediated via, at least in part, GPR41. Thus, these results suggest that GPR41 may play an important role in improving insulin sensitization for the management of type 2 diabetes and related complications.
